# Prolonged Continuous Theta Burst Stimulation to Demonstrate a Larger Analgesia as Well as Cortical Excitability Changes Dependent on the Context of a Pain Episode

**DOI:** 10.3389/fnagi.2021.804362

**Published:** 2022-01-28

**Authors:** Ying Liu, Lina Yu, Xianwei Che, Min Yan

**Affiliations:** ^1^Department of Anesthesiology, The Second Affiliated Hospital of Zhejiang University School of Medicine, Hangzhou, China; ^2^Centre for Cognition and Brain Disorders, The Affiliated Hospital of Hangzhou Normal University, Hangzhou, China; ^3^Institute of Psychological Sciences, Hangzhou Normal University, Hangzhou, China

**Keywords:** pcTBS, pain, motor-evoked potential, cortical silent period, cortical excitability

## Abstract

A series of neuropathic pain conditions have a prevalence in older adults potentially associated with declined functioning of the peripheral and/or central nervous system. Neuropathic pain conditions demonstrate defective cortical excitability and intermissions, which raises questions of the impact of pain on cortical excitability changes and when to deliver repetitive transcranial magnetic stimulation (rTMS) to maximize the analgesic effects. Using prolonged continuous theta-burst stimulation (pcTBS), a relatively new rTMS protocol to increase excitability, this study was designed to investigate pcTBS analgesia and cortical excitability in the context of pain. With capsaicin application, twenty-nine healthy participants received pcTBS or Sham stimulation either in the phase of pain initialization (capsaicin applied) or pain ascending (20 min after capsaicin application). Pain intensity was measured with a visual-analogic scale (VAS). Cortical excitability was assessed by motor-evoked potential (MEP) and cortical silent period (CSP) which evaluates corticospinal excitability and GABAergic intracortical inhibition, respectively. Our data on pain dynamics demonstrated that pcTBS produced a consistent analgesic effect regardless of the time frame of pcTBS. More importantly, pcTBS delivered at pain initialization induced a larger pain reduction and a higher response rate compared to the stimulation during pain ascending. We further provide novel findings indicating distinct mechanisms of pcTBS analgesia dependent on the context of pain, in which pcTBS delivered at pain initialization was able to reverse depressed MEP, whereby pcTBS during pain ascending was associated with increased CSP. Overall, our data indicate pcTBS to be a potential protocol in pain management that could be delivered before the initialization of a pain episode to improve rTMS analgesia, potentially through inducing early corticospinal excitability changes that would be suppressed by nociceptive transmission.

## Introduction

Chronic neuropathic pain results from etiologically diverse disorders affecting the peripheral or the central nervous system (Scholz et al., 2019). A series of neuropathic pain conditions (e.g., postherpetic neuralgia and diabetic neuropathy) has a prevalence in older adults (Cunningham et al., 2016; John and Canaday, 2017; Ponirakis et al., 2019). Transcranial magnetic stimulation (TMS) is a safe and noninvasive form of brain stimulation. Repetitive TMS (rTMS) can induce neuroplastic changes, which have been used to manage chronic pain (Mhalla et al., 2010; Klein et al., 2015; Parker et al., 2016) and other neural degenerative conditions (Ahmed et al., 2012; Rabey et al., 2013). Indeed, high-frequency (≥5 Hz) rTMS over the primary motor cortex (M1) is suggested to be able to reduce neuropathic pain in randomized controlled studies (Hosomi et al., 2013, 2020; Attal et al., 2021). Overall, the clinical application of rTMS in chronic pain is still limited by the response rate, whereby it is close to moderate and far from being excellent at its best (Lefaucheur et al., 2014). It is therefore important to optimize the analgesic efficacy of rTMS.

In clinical settings, there is significant variability in pain intensity between rTMS treatment sessions. More specifically, the progress of a pain episode is associated with pain initialization, pain ascending and stabilizing, and pain reduction gradually. This raises questions of the temporal dynamics of pain, its impact on cortical excitability, and when to deliver treatments to maximize the benefits. Meta-analytic evidence has indicated shortened cortical silent period (CSP) in chronic pain populations (Parker et al., 2016), in which the duration of CSP is thought to indicate GABA_B_-mediated intracortical inhibition (Werhahn et al., 1999). Moreover, motor-evoked potential (MEP) has been repeatedly demonstrated to be suppressed by both provoked (Farina et al., 2001) and chronic pain (Cosentino et al., 2014; Coppola et al., 2019). These studies demonstrate an inhibitory impact of pain on corticospinal excitability and intracortical inhibition. More importantly, the analgesic effects of rTMS could result from the restoration of defective cortical excitability induced by pain (Lefaucheur et al., 2006; Mhalla et al., 2011). It is therefore important to systemically investigate the analgesic influence of rTMS in the context of pain levels and cortical excitability changes.

Neuropathic pain syndromes are clinically characterized by spontaneous pain which has obvious intermissions. Topical application of capsaicin demonstrates temporal dynamics of pain. In general, pain perception tended to reach a significant level after 20 min of capsaicin application, reached the peak amplitude after 40 min and stabilized for at least 20 min, and then started the descending process from 70 to 80 min onward (Farina et al., 2001; Fierro et al., 2010). This pattern of pain dynamics is suggested to be able to mimic the progress of a pain episode (Frias and Merighi, 2016), which provides a unique opportunity to investigate the analgesic impact of rTMS at different pain levels particularly for neuropathic pain conditions.

The investigation of rTMS protocols is also important for improving rTMS analgesia. Theta-burst stimulation (TBS) mimics the bursts of neuronal firing which results in robust long-term potentiation, that is, the combination of the complex-spike pattern (∼100 Hz) with a theta frequency (∼5 Hz) repetition rate (Larson et al., 1986). Continuous TBS (cTBS) is designed to decrease excitability (Huang et al., 2005), whereby prolonged cTBS (pcTBS, i.e., multiple cTBS being delivered continuously) has recently been demonstrated to increase excitability (Klirova et al., 2020; McCalley et al., 2021). Specifically, pcTBS with two times the duration of cTBS converted the conventional inhibitory effect into a facilitatory one by means of increased MEP (Klirova et al., 2020). More importantly, pcTBS was found to have comparable (De Martino et al., 2019) or even better (Moisset et al., 2015) analgesic effects than standard 10 Hz rTMS (but see Klirova et al., 2020). These findings together call for more studies to validate the analgesic efficacy of pcTBS.

Using the fast and patterned pcTBS protocol, the current study was designed to investigate rTMS analgesia in the context of pain levels. With capsaicin application, participants received pcTBS or Sham stimulation either in the phase of pain initialization or pain ascending. These time frames were adopted as TMS tends to take time to act on cortical excitability (Di Lazzaro et al., 2010; Qiu et al., 2020) and therefore it might be late when the pain reaches peak amplitude. MEP and CSP were also evaluated with the purpose of examining cortical excitability dynamics modulated by both pain and pcTBS. It is hypothesized that pcTBS delivered either in the initialization or the ascending phase of pain would be able to reduce pain experience and to increase MEP and CSP compared to the Sham stimulation. We further sought to compare the analgesic efficacy of pcTBS in different pain contexts and to explore the associated cortical excitability changes.

## Materials and Methods

### Participants

An *a priori* sample size calculation (α = 0.05, power = 0.8, effect size = 0.3) indicated a minimum of 24 participants for the study to be sufficiently powered. The effect size of 0.3 was estimated based on the literature using the same stimulation protocol (Klirova et al., 2020). A group of 29 healthy, right-handed, TMS eligible (Rossi et al., 2011) adults were recruited to account for potential dropouts. Exclusion criteria included history or current diagnosis of psychiatric disorder, or use of psychoactive medication, as assessed by the Mini International Neuropsychiatric Interview (MINI) (Sheehan et al., 1998). No participant withdrew from this study, data from 29 participants (age range: 18–65 years, mean ± SD: 27.17 ± 12.44, 15 females) were therefore analyzed. All participants provided written informed consent before study commencement. This study was approved by the Ethics Committee in the Centre for Cognition and Brain Disorders of Hangzhou Normal University (20210330) and was conducted in accordance with the Declaration of Helsinki.

### Experimental Design and Procedure

**FIGURE 1:**
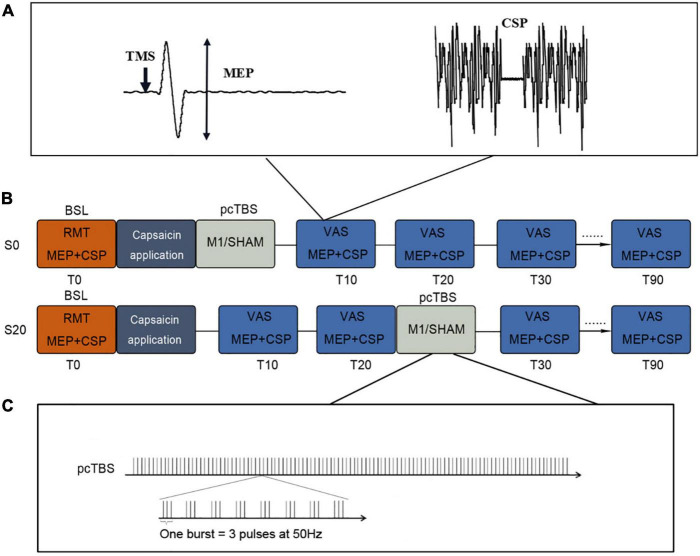
Study design of this study. **(A)** MEP and CSP protocols. **(B)** Study protocol. pcTBS was delivered immediately (S0) or 20 min (S20) after capsaicin application. Sham stimulation was randomized to these two conditions at a 50% chance. Pain intensity (VAS) and cortical excitability (MEP and CSP) were evaluated every 10 min up to 90 min. **(C)** pcTBS protocol. pcTBS consists of a burst of 3 pulses given at 50 Hz repeated every 5 Hz, totaling 1,200 consecutive pulses. RMT, resting motor threshold; MEP, motor-evoked potential; CSP, cortical silent period; VAS, visual analog scale.

This was a single-blind, crossover, Sham-controlled study (Figure 1). Participants visited the lab three times (≥72 h intervals), receiving a single session of pcTBS in the ascending (S20) or initialization (S0) of pain, or Sham stimulation (the same as pcTBS protocol, with the coil being flipped 90° to the scalp) with the sequence being pseudo randomized and counterbalanced. In each session, participants were exposed to pain induced by capsaicin application. Corticospinal excitability was measured with MEP and CSP before and after pcTBS in 90 min at an interval of 10 min.

### Resting Motor Threshold and Corticospinal Excitability

Each session started with the assessment of the resting motor threshold (RMT). RMT was defined as the minimum intensity to induce MEPs > 0.05 mV of the first dorsal interosseous (FDI) muscle in 5/10 trials. Single pulses to the hand region of the left M1 (45° to the midline, handle pointing backward) at 5 s ± 10% jitter intervals were sent by a figure-eight coil connected to a Magstim Rapid^2^ system (Magstim Company Ltd., United Kingdom). Coil position was measured relative to the nasion and inion to facilitate consistent re-positioning of the coil between sessions (Che et al., 2019).

Corticospinal excitability was measured with MEP and CSP at rest and during a sustained voluntary FDI muscle contraction, respectively (Hupfeld et al., 2020). The maximal voluntary contraction (MVC) was calculated and 20% of MVC was used for tonic contraction in CSP (Fling and Seidler, 2012). A total of 40 single pulses (20 of each) were consecutively delivered to the hand region of the left M1 at 120% RMT (45° to the midline, handle pointing backward). It is worth noting that CSP was evaluated following MEP as the muscle contraction during CSP may have an impact on MEP (Conforto et al., 2004). Corticospinal excitability was measured before and after pain and pcTBS in 90 min at an interval of 10 min, with the purpose to capture the dynamics of corticospinal excitability modulated by pain and pcTBS.

### Pain Protocol

Capsaicin application is a widely used tonic pain protocol that has been demonstrated to evoke tonic heat pain lasting up to 90 min (Farina et al., 2001; Fierro et al., 2010). In this study, capsaicin (Dolpyc Teofarma 1%) was applied over the dorsal surface of the right hand in an area of 2 × 2 cm. Pain experience was measured by using a 0–10 point visual-analogic scale (VAS) (0: no pain, 1–3: mild pain, 4–6: moderate pain, and 7–10: severe pain) in 90 min at an interval of 10 min. Pain measurements were collected whereas corticospinal excitability was evaluated for consistency.

### Repetitive Transcranial Magnetic Stimulation

Prolonged continuous theta-burst stimulation (pcTBS) was administered to the left M1 at 80% RMT, consisting of a burst of 3 pulses given at 50 Hz repeated every 5 Hz (Moisset et al., 2015; De Martino et al., 2019). A total of 1,200 pulses were delivered with the TMS coil positioned in a posterior-anterior (PA) direction parallel to the midline. In a pain initialization session (S0), pcTBS was delivered immediately after capsaicin application. Meanwhile, a pain ascending session (S20) delivered pcTBS 20 min after capsaicin application. This time point falls in the middle of the pain ascending phase which in general lasts for 40 min (Farina et al., 2001; Brighina et al., 2011; Mavromatis et al., 2016). The Sham stimulation was delivered using the same pcTBS protocol, with the coil being flipped 90° to the scalp so that the magnetic field would be delivered away from the scalp (Pascual-Leone et al., 1999). It is noted that the Sham stimulation was randomized to these two conditions (S0 or S20) at a 50% chance.

The side effect was assessed with painful sensations at the end of each session (Klein et al., 2015), using a numerical rating scale where 0 indicates no pain and 10 most intensive pain.

### Data Analysis

Motor-evoked potential (MEP) was calculated from peak to peak. The calculation of CSP duration was based on the mean consecutive difference (MCD) by Garvey et al. (2001), which was highly recommended in a recent review (Hupfeld et al., 2020). This method is briefly described here: (1) all silent period trials were rectified using the absolute value and then were averaged; (2) the MCD of 100 ms of prestimulus EMG was calculated, in which the MCD is the mean successive difference between individual data points; (3) thresholds were set at ±MCD × 2.66 (i.e., 3 SDs), which covers 99.76% of possible prestimulus EMG data points; (4) silent period onset was determined as the time point at which the poststimulus EMG falls below the variation threshold for three consecutive data points, while the silent period offset was defined as the point at which the poststimulus EMG returns above the variation threshold for three consecutive data points.

### Statistical Analyses

Statistical analyses were performed with SPSS (version 22; IBM Corporation, Armonk, NY, United States). We initially checked the normality of pain ratings in different combinations of our two factors (i.e., condition and time) with the Shapiro–Wilk test. The normality test was not violated (*P* > 0.05). A repeated measure two-way ANOVA was performed on pain ratings, with condition (S0, S20, and Sham) and time (T0–T10) being specified as within factors. *Post-hoc* pairwise comparisons were conducted to further explore the significant main and interaction effects, with the α-level set to 0.05 and Bonferroni corrected.

As pain ratings demonstrated a clear pattern of baseline (T0), ascending (T10–T30), stabilizing (T40–T60), and descending (T70–T90) phases (please refer to Results), pain ratings were further averaged within each phase for clarity. Pain ratings were then analyzed using a 3 (condition: S0, S20, and Sham) × 4 (time: baseline, ascending, stabilizing, and descending) repeated measures ANOVA and Bonferroni corrected at 0.05.

Motor-evoked potential and CSP data were also analyzed based on this pattern using three conditions: (S0, S20, and Sham) × 4 (time: baseline, ascending, stabilizing, and descending) two-way ANOVAs.

**FIGURE 2:**
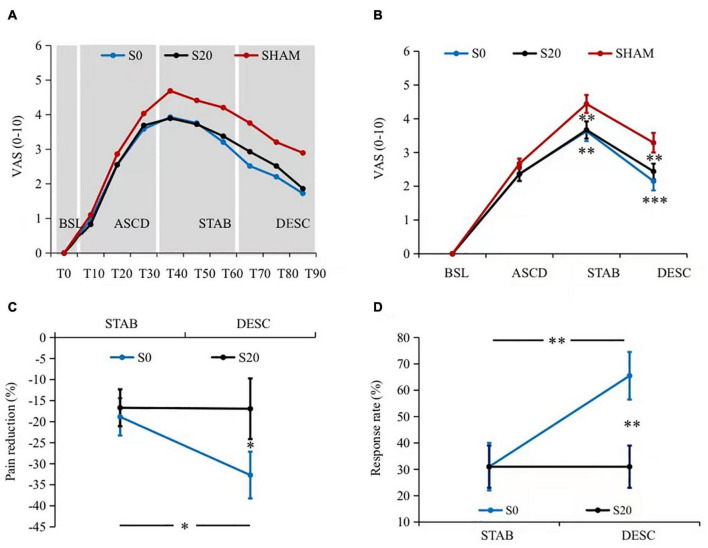
Pain intensity results. **(A)** The dynamics of pain perception. Pain ratings demonstrated a clear pattern of baseline (T0), ascending (T10–T30, all *P*_corrected_ < 0.05 compared to the predecessor), stabilizing (T40–T60, all *P*_corrected_ > 0.05 compared to the predecessor), and descending (T70–T90, all *P*_corrected_ < 0.05 compared to the peak amplitude T40, and all *P*_corrected_ < 0.05 compared to the predecessor) phases. In addition, both the S0- and S20-pcTBS decreased pain from T40 to T90 compared to the Sham stimulation. **(B)** The averaged pain ratings within each phase. Similarly, S0- and S20-pcTBS decreased pain in the stabilizing (all *P*_corrected_ < 0.05) and descending (all *P*_corrected_ < 0.05) stages compared to the Sham stimulation. **(C)** The analgesic efficacy of S0- and S20-pcTBS. S0-pcTBS resulted in a larger pain reduction in the descending phase compared to the S20-pcTBS and that in the stabilizing phase. **(D)** The results of analgesic efficacy in terms of response rate. S0-pcTBS had a higher response rate in the descending phase compared to the S20-pcTBS and that in the stabilizing phase. *, ^**^, and ^***^ denote *P* < 0.05, *P* < 0.01, and *P* < 0.001 compared to the Sham. BSL, baseline; ASCD, ascending; STAB, stabilization; DESC, descending. Please refer to the Supplementary Material for figures with all the samples and variances.

As the S0- and S20-stimulation tended to show a difference in analgesic efficacy (Figures 2A,B), we further compared the analgesic efficacy of these two conditions. Pain reduction of S0- and S20-stimulation was derived relative to the Sham stimulation [e.g., (S0_DESC_ – Sham_DESC_)/S0_DESC_ × 100%]. We also compared the response rate which was determined by no less than 30% in pain reduction (based on the formula of pain reduction above) in TMS literature (Dworkin et al., 2008). A two-way repeated-measures ANOVA (condition: S0, S20, and Sham; time: stabilizing and descending) and chi-squared (χ^2^) statistic were performed on pain reduction and response rate, respectively. It is worth noting that the McNemar test was used for χ^2^ statistics which was specifically designed for binary dependent variables in χ^2^ statistics (Agresti, 2003). We also performed a series of correlation analyses to establish the relationship between pain reduction and motorcortical excitability changes. As the participants were from differing age groups, we initially examined the relationship between age and pain/MEP/CSP. Changes in pain ratings, MEP, and CSP at each phase were calculated relative to the Sham stimulation. Bivariate or partial (when age had a significant relationship with at least one of the variables) correlations were conducted between changes in pain ratings, MEP, and CSP. We also explored the MEP-to-CSP ratio as it has been used in previous studies (Terada et al., 2016; Latella et al., 2018).

## Results

Our data indicated that no participants reported painful sensations by the end of each session.

### Effects of Prolonged Continuous Theta-Burst Stimulation on Pain Experience

All of the participants reported no pain at baseline as the capsaicin application took minutes to induce pain. Our data on pain ratings demonstrated a similar pattern of temporal dynamics to the literature. There was a significant time effect on pain ratings (*F*_2.83,79.19_ = 63.22, *P*_*corrected*_ = 0.001, $\eta_{p}^{2}$ = 0.69) (Figure 2A). Pairwise comparisons of pain dynamics demonstrated statistically different stages characterized by baseline (T0), ascending (T10–T30, all *P*_*corrected*_ < 0.05 compared to the predecessor), stabilizing (T40–T60, all *P*_*corrected*_ > 0.05 compared to the predecessor), and descending (T70–T90, all *P*_*corrected*_ < 0.05 compared to the peak amplitude T40, and all *P*_*corrected*_ < 0.05 compared to the predecessor) phases. There was also a significant condition × time interaction effect on pain ratings (*F*_5.89,164.78_ = 2.38, *P*_*corrected*_ = 0.032, $\eta_{p}^{2}$ = 0.08) (Figure 2A). Pairwise comparisons indicated that pcTBS at S0 (all *P*_*corrected*_ < 0.05) and S20 (all *P*_*corrected*_ < 0.05) decreased pain perception compared to the Sham stimulation from T40 onward to T90, i.e., in the stabilizing and descending phase.

A 3 (condition) × 4 (time) repeated measures ANOVA revealed a significant condition × time interaction effect on pain (*F*_3.89,108.78_ = 5.19, *P_*corrected*_* = 0.001, $\eta_{p}^{2}$ = 0.16) (Figure 2B and Table 1). Pairwise comparisons indicated that both the S0- and S20-pcTBS decreased pain in the stabilizing (S0: *P*_*corrected*_ = 0.003, S20: *P*_*corrected*_ = 0.001) and descending (S0: *P*_*corrected*_ = 0.000, S20: *P*_*corrected*_ = 0.007) phase compared to the Sham stimulation (S0: Mean_STAB_ = 3.63, Mean_DESC_ = 2.15; S20: Mean_STAB_ = 3.67, Mean_DESC_ = 2.44; Sham: Mean_STAB_ = 4.44, Mean_DESC_ = 3.29).

**TABLE 1:** Summaries of ANOVA results.

Source	*df*	Mean square	*F*	*p*	$\eta_{p}^{2}$
***(VAS)***					
Condition	2	10.693	18.808	0.000***	0.402
Time	2.222	313.584	109.085	0.000***	0.796
Condition x Time	3.885	3.275	5.189	0.001**	0.156
***(MEP)***					
Condition	1.435	2.097	4.379	0.030*	0.135
Time	2.302	0.154	1.524	0.223	0.052
Condition x Time	2.963	0.368	2.895	0.041*	0.094
***(CSP)***					
Condition	1.453	1203.57	3.589	0.050*	0.114
Time	1.795	201.016	2.544	0.094	0.083
Condition x Time	3.624	170.500	2.518	0.050*	0.082

VAS, visual-analogic scale; MEP, motor evoked potential; CSP, cortical silent period; df, degrees of freedom. *P < 0.05; **P < 0.01; ***P < 0.001.

Our data on the analgesic efficacy indicated a significant condition × time interaction effect on pain (*F*_1,28_ = 4.86, *P_*corrected*_* = 0.036, $\eta_{p}^{2}$ = 0.15) (Figure 2C). Pairwise comparisons indicated that S0-pcTBS (Mean_DESC_ = −32.69%) resulted in a larger pain reduction (*P*_*corrected*_ = 0.048) in the descending phase compared to the S20 (Mean_DESC_ = −16.92%). Pain reduction in the descending phase (Mean_DESC_ = −32.69%) was also larger (*P*_*corrected*_ = 0.030) than that in the stabilization stage (Mean_STAB_ = −18.84%) induced by S0-pcTBS. The response rate data indicated a higher response rate (*P*_*corrected*_ = 0.006) in the S0 (Mean_DESC_ = 65.5%, 19 responders) compared to the S20 condition (Mean_DESC_ = 31%, 9 responders) in the descending phase (Figure 2D). The response rate was also higher (*P*_*corrected*_ = 0.006) in the descending phase (Mean_DESC_ = 65.5%, 19 responders) compared to the stabilization stage (Mean_DESC_ = 31%, 9 responders) in the S0 stimulation.

### Effects of Repetitive Transcranial Magnetic Stimulation on Motorcortical Excitability

**FIGURE 3:**
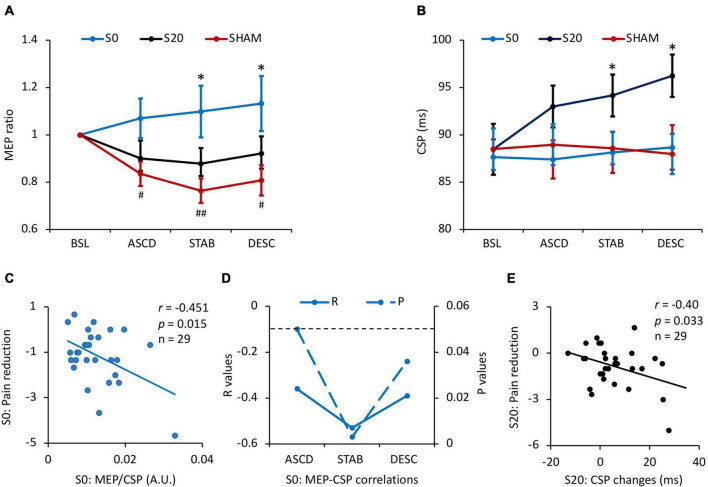
Results of motorcortical excitability and the associations with pain reduction. **(A)** The effects of stimulation on MEP. Pain inhibited MEP in all stages compared to the baseline (all *P*_*corrected*_ < 0.05) in the Sham condition, while pcTBS at S0 reversed depressed MEP in the stabilizing (*P*_*corrected*_ = 0.022) and descending stages (*P*_*corrected*_ = 0.024), and a trend increase in the ascending phase (*P*_*corrected*_ = 0.06). **(B)** The effects of pcTBS on CSP. CSP duration was increased by the S20-pcTBS in the stabilizing (*P*_*corrected*_ = 0.033) and descending (all *P*_*corrected*_ = 0.015) stages compared to the Sham stimulation. **(C)** A significant negative correlation between the early (ascending) MEP/CSP ratio and late (descending) pain reduction in S0-pcTBS. **(D)** The dynamic correlations between MEP and CSP changes in the S0-pcTBS. MEP changes in the early phase (ascending) was negatively associated with CSP changes in the ascending, stabilization, and descending phases. **(E)** A significant negative correlation between the early (stabilization) CSP changes and late (descending) pain reduction in S20-pcTBS. * represents *P* < 0.05 compared to Sham; # and ## represent P < 0.05 and P < 0.01 compared to the baseline. A.U. denotes arbitrary unit. The dash line denotes P < 0.05. Error bars represent mean ± SE; BSL, baseline; ASCD, ascending; STAB, stabilization; DESC, descending. All the correlations are presented with age regressed as a covariate.

There was a condition × time interaction effect on MEP (*F*_2.96,82.96_ = 2.90, *P_*corrected*_* = 0.041, $\eta_{p}^{2}$ = 0.09) (Figure 3A and Table 1). Pairwise comparisons indicated that capsaicin-induced pain significantly reduced MEP amplitude in all phases (ASCD: *P*_*corrected*_ = 0.023; STAB: *P*_*corrected*_ = 0.001; DESC: *P*_*corrected*_ = 0.033) compared to the baseline in the Sham condition (Mean_ASCD_ = 0.84, Mean_STAB_ = 0.76, Mean_DESC_ = 0.81). Meanwhile, pcTBS at S0 resulted in larger MEPs in the stabilizing (*P*_*corrected*_ = 0.022) and descending stages (*P*_*corrected*_ = 0.024), and a trend increase in the ASCD phase (*P*_*corrected*_ = 0.06) compared to the Sham stimulation (S0: Mean_ASCD_ = 1.07, Mean_STAB_ = 1.10, Mean_DESC_ = 1.13; Sham: Mean_ASCD_ = 0.84, Mean_STAB_ = 0.76, Mean_DESC_ = 0.81). No significant difference was found between the S0 and S20 conditions (*P*_*corrected*_ = 0.38).

In terms of CSP, there was a significant condition × time interaction effect (*F*_3.62,101.47_ = 2.52, *P*_*corrected*_ = 0.050, $\eta_{p}^{2}$ = 0.082) (Figure 3B and Table 1). Pairwise comparisons indicated that pcTBS at S20 resulted in a larger CSP in the stabilizing (*P*_*corrected*_ = 0.022) and descending (*P*_*corrected*_ = 0.015) stages compared to the Sham stimulation (S20: Mean_STAB_ = 94.16, Mean_DESC_ = 96.24; Sham: Mean_STAB_ = 88.59, Mean_DESC_ = 87.98). No other significant differences were found between conditions or time (all *P*_*corrected*_ > 0.05).

### Relationship Between Pain Reduction and Cortical Excitability Changes

There was a significant negative correlation between age and baseline CSP across three sessions (*r* = −0.42, *p* = 0.02). We therefore regressed age in the following correlation analyses. We found a significant negative correlation between the early (ascending phase) MEP/CSP ratio and late (descending phase) pain reduction in S0-pcTBS (*r* = −0.45, *p* = 0.015) (Figure 3C). Moreover, MEP changes in the early phase (ascending) was negatively associated with CSP changes in the ascending (*r* = −0.39, *p* = 0.039), stabilization (*r* = −0.53, *p* = 0.004), and descending (*r* = −0.39, *p* = 0.038) phases in the S0 stimulation (Figure 3D). In terms of S20 stimulation, there was a significant negative correlation (*r* = −0.40, *p* = 0.033) between the early (stabilization) CSP increasement and late (descending) pain reduction (Figure 3E).

## Discussion

Using the fast and patterned pcTBS protocol, this study was designed to investigate rTMS analgesia in the context of pain. Our data demonstrated a consistent analgesic effect of pcTBS. More importantly, pcTBS delivered at pain initialization induced a larger pain reduction and a higher response rate compared to the stimulation during pain ascending. We also provide novel findings indicating distinct mechanisms of pcTBS analgesia in the context of pain. pcTBS delivered in the phase of pain initialization was able to reverse depressed MEP, whereby pcTBS in the ascending phase of pain was associated with increased CSP.

Our data on pain dynamics demonstrated obvious phases of capsaicin-induced pain, (Farina et al., 2001; Fierro et al., 2010) which provides a unique opportunity to investigate the analgesic impact of rTMS in different phases of a pain episode among chronic pain patients. More importantly, our data demonstrated the consistency of pcTBS analgesia regardless of the time frame to deliver stimulation. Due to the capacity to increase cortical excitability in a short period of time (Klirova et al., 2020; McCalley et al., 2021), studies have begun to evaluate the effect of pcTBS in pain management (Moisset et al., 2015; De Martino et al., 2019; Klirova et al., 2020). Our findings provide direct evidence to support pcTBS analgesia, which is critical for pcTBS to be used in the optimization of rTMS analgesia.

It is important to highlight that pcTBS delivered at pain initialization induced larger analgesia compared to the stimulation during pain ascending, which was characterized by a larger pain reduction and a higher response rate in the descending phase of pain. This is important to many chronic pain conditions as it provides insights on when to deliver treatments to maximize rTMS analgesia. As discussed earlier, neuropathic pain conditions are characterized by clear pain intermissions. Our findings indicate that receiving rTMS before pain initialization or more broadly at the early phase of a pain attack may be able to achieve larger analgesia. However, one needs to be cautious on this conclusion as these two stimulation conditions were close in time and demonstrated similar patterns of analgesia. Nonetheless, we provide evidence to demonstrate dynamic cortical excitability changes associated with the superior analgesic efficacy in the pain initialization condition (see below discussions on excitability mechanisms).

Prolonged continuous theta-burst stimulation (pcTBS) given at different phases of a pain episode may be associated with distinct cortical excitability changes. Our data indicated that pain induction resulted in a significant decrease in MEP amplitude in the absence of pcTBS intervention. This finding is consistent with previous studies in which MEP amplitude was reduced by chronic pain (Lefaucheur et al., 2006; Cosentino et al., 2014; Rittig-Rasmussen et al., 2014; Parker et al., 2016). Reduced motor cortical output is associated with the imbalance of neurotransmission in the central nervous system evoked by the ascending transmission of nociception. Meanwhile, pcTBS delivered during pain initialization increased MEP amplitude from the ascending to the stabilizing and descending stages. Moreover, this pattern of MEP changes aligns nicely with the dynamics of analgesia. In addition, increased “excitation-to-inhibition” ratio (i.e., MEP/CSP) in the early phase of pain was associated with a larger analgesic effect in the late stage of pain induction (Figure 3C), providing further evidence to support a mechanism of motorcortical excitability associated with pcTBS analgesia. However, pcTBS delivered in the ascending phase of pain had no impact on MEP amplitude, which indicates the involvement of other mechanisms than motorcortical excitation.

Indeed, pcTBS delivered in the ascending phase of pain enhanced CSP especially in pain stabilizing and descending phases (Figure 3B). The cortical silent period is thought to reflect the activity of GABA_B_-mediated inhibitory circuits acting upon the corticospinal pathway (Siebner et al., 1998; Werhahn et al., 1999). These changes in CSP are in line with significant pain reduction during these two phases which took place from 20 min poststimulation onward. It is worth noting that MEP amplitude was significantly reduced by capsaicin application by this time (see Sham condition). pcTBS may therefore act on GABA_B_-mediated intracortical inhibition to reduce pain. Indeed, the balance between cortical excitation and inhibition tends to be disrupted by chronic pain conditions (Barr et al., 2013), and chronic pain is associated with reduced intracortical inhibition (Parker et al., 2016). More importantly, two studies have demonstrated the capacity of rTMS to reverse defective intracortical inhibition in chronic pain patients (Lefaucheur et al., 2006; Mhalla et al., 2011). Our results also indicated that early (i.e., stabilization) CSP increment was associated with a larger pain reduction in the late phase (i.e., descending) (Figure 3E). We, therefore, provide the first line of evidence that a single session of pcTBS is sufficient to increase GABA_B_-mediated intracortical inhibition which is associated with decreased pain.

There were no significant changes in CSP when pcTBS was delivered during the initialization of pain (Figure 3C). It is possible that increased motorcortical excitability as indexed by MEP is sufficient to produce pain analgesia. MEP changes in the ascending phase of pain were negatively associated with CSP changes in the ascending, stabilization, and descending phases of pain when pcTBS was delivered during pain initialization. These findings are also consistent with the balance of cortical excitation and inhibition whereby pcTBS during pain initialization may drive early cortical excitation to reduce pain. Overall, we provide interesting findings to indicate superior analgesia when pcTBS is delivered before pain initialization or at the early phase of a pain attack in a broader way. Moreover, this effect is associated with early motorcortical excitability changes which may work against depressed MEP caused by nociceptive transmission. Otherwise, pcTBS may act on the alternative GABA_B_-mediated intracortical inhibition to modulate the corticospinal pathway when pain stabilized.

There were some limitations in the study. We delivered pcTBS in the initialization and ascending phases of a pain episode without modeling the pain stabilization phase. This was designed as rTMS tends to take time to act, as demonstrated by changes in cortical excitability and pain perception in our data. We averaged data in each phase of pain, especially for the ascending phase whereby pcTBS was delivered right in between, to simplify the profiles of pain dynamic and to highlight the stage effect. Although our data demonstrated the same analgesia between these two methodologies, data presentation of each time point would also be appreciated. The M1 was located using the hotspot methodology. Although the hotspot approach was considered as an effective and efficient method to locate the M1 (Lefaucheur and Nguyen, 2019), a neuronavigation system is able to assist localization and the identification of disease-relevant brain connections and networks mediating positive treatment outcomes (Cash et al., 2020).

Our findings may bear significance for the clinical application of rTMS in pain management. Our data demonstrate a consistent analgesic effect of pcTBS regardless of the context of pain. pcTBS, therefore, represents a potential protocol for pain management that has not been evaluated in chronic pain populations. Moreover, our data demonstrated superior analgesia when pcTBS is delivered at the early compared to the ascending phase of a pain attack. This finding provides direct evidence to optimize rTMS analgesia in terms of when to deliver rTMS treatments. Besides, multiple sessions of pcTBS could be delivered within a single day due to its efficiency and efficacy, with the purpose to accelerate standard rTMS treatment protocols (Blumberger et al., 2018). In addition, our findings indicate distinct mechanisms of pcTBS analgesia when it is delivered at different phases of a pain episode. These findings provide insight for optimizing pcTBS analgesia in which pcTBS protocols can be designed to improve motorcortical excitability or intracortical inhibition dependent on the phases of a pain episode in a treatment session. Findings from this study provide insights on healthy aging and on the management of age-related neurodegenerative conditions. In one way, we demonstrated pcTBS to be able to reduce pain. This is important to healthy aging as a significant portion of old adults suffer from pain conditions (Jones et al., 2016; Sherman et al., 2020) and a range of neuropathic pain conditions (e.g., postherpetic neuralgia and diabetic neuropathy) have a prevalence in older adults (Cunningham et al., 2016; John and Canaday, 2017; Ponirakis et al., 2019). In another way, we provided neuroplastic changes underlying the analgesic effect of pcTBS. These findings add to our understanding of how rTMS can be used to manage age-related neurodegenerative conditions through neuroplastic mechanisms. In addition, our findings on pcTBS analgesia represent an optimizing effort of rTMS efficacy which has clear implications for age-related neurodegenerative conditions such as Alzheimer’s disease whereby rTMS has a limited effect (Lefaucheur et al., 2014).

To conclude, this study demonstrated a consistent analgesic effect of pcTBS, which could be delivered before the initialization of a pain episode to improve rTMS analgesia. Moreover, this effect is associated with early motorcortical excitability changes which may work against depressed MEP caused by nociceptive transmission.

## Data Availability Statement

All data generated and analyzed during the current study will be available from the first author on reasonable request. Requests to access these datasets should be directed to YL, 350373328@qq.com.

## Ethics Statement

The studies involving human participants were reviewed and approved by the Ethics Committee in the Centre for Cognition and Brain Disorders of Hangzhou Normal University (20210330) and was conducted in accordance with the Declaration of Helsinki. The patients/participants provided their written informed consent to participate in this study.

## Author Contributions

XC and YL contributed to data acquisition and statistical analysis. YL was a major contributor in writing the manuscript. XC reviewed and edited the manuscript. All authors contributed to the conception and design of the study, and read as well as approved the final manuscript.

## Conflict of Interest

The authors declare that the research was conducted in the absence of any commercial or financial relationships that could be construed as a potential conflict of interest.

## Publisher’s Note

All claims expressed in this article are solely those of the authors and do not necessarily represent those of their affiliated organizations, or those of the publisher, the editors and the reviewers. Any product that may be evaluated in this article, or claim that may be made by its manufacturer, is not guaranteed or endorsed by the publisher.

## Supplementary Material

Supplementary Figure 1 Results of all samples and variances. **(A)** Shows the samples and variances of the averaged pain ratings within each phase. Similarly, S0- and S20-pcTBS decreased pain in the stabilizing (all *P*_*corrected*_ < 0.05) and descending (all *P*_*corrected*_ < 0.05) stages compared to the Sham stimulation. **(B)** Indicates the samples and variances of analgesic efficacy of S0- and S20-pcTBS. S0-pcTBS resulted in a larger pain reduction in the descending phase compared to the S20-pcTBS as well as that in the stabilizing phase. **(C)** Shows the samples and variances of MEP. Pain inhibited MEP in all stages compared to the baseline (all *P*_*corrected*_ < 0.05) in the Sham condition, while pcTBS at S0 reversed depressed MEP in the stabilizing (*P*_*corrected*_ = 0.022) and descending stages (*P*_*corrected*_ = 0.024), as well as a trend increase in the ascending phase (*P*_*corrected*_ = 0.06). **(D)** Shows the samples and variances of pcTBS on CSP. CSP duration was increased by the S20-pcTBS in the stabilizing (*P*_*corrected*_ = 0.033) and descending (all *P*_*corrected*_ = 0.015) stages compared to the Sham stimulation.
