# Supplementary figures and images for: Prolonged Continuous Theta Burst Stimulation to Demonstrate a Larger Analgesia as Well as Cortical Excitability Changes Dependent on the Context of a Pain Episode
Source: Front Aging Neurosci. 2022 Jan 28;13:804362. doi: 10.3389/fnagi.2021.804362 (PMC8833072; doi:10.3389/fnagi.2021.804362)

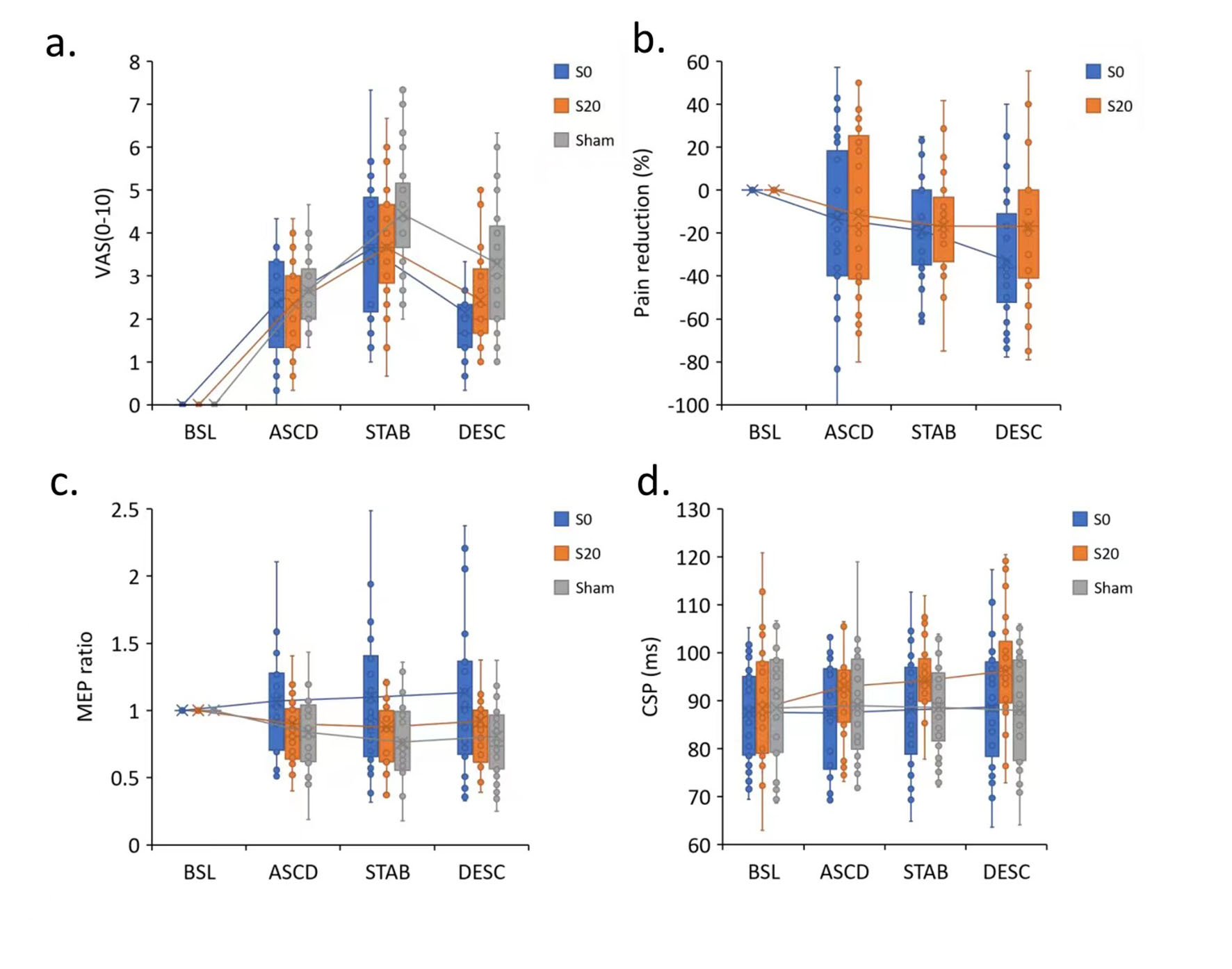

Supplement: Supplementary Figure 1 — Results of all samples and variances. (A) Shows the samples and variances of the averaged pain ratings within each phase. Similarly, S0- and S20-pcTBS decreased pain in the stabilizing (all Pcorrected < 0.05) and descending (all Pcorrected < 0.05) stages compared to the Sham stimulation. (B) Indicates the samples and variances of analgesic efficacy of S0- and S20-pcTBS. S0-pcTBS resulted in a larger pain reduction in the descending phase compared to the S20-pcTBS as well as that in the stabilizing phase. (C) Shows the samples and variances of MEP. Pain inhibited MEP in all stages compared to the baseline (all Pcorrected < 0.05) in the Sham condition, while pcTBS at S0 reversed depressed MEP in the stabilizing (Pcorrected = 0.022) and descending stages (Pcorrected = 0.024), as well as a trend increase in the ascending phase (Pcorrected = 0.06). (D) Shows the samples and variances of pcTBS on CSP. CSP duration was increased by the S20-pcTBS in the stabilizing (Pcorrected = 0.033) and descending (all Pcorrected = 0.015) stages compared to the Sham stimulation. [file Image_1.JPEG]
